# Screen time, physical activity, and overweight/obesity among school-aged children and adolescents in Heilongjiang Province, China: a cross-sectional study

**DOI:** 10.3389/fpubh.2026.1959738

**Published:** 2026-10-28

**Authors:** Xiaoyu Zhao, Zixuan Ru, Yanpeng Xie, Haojing Li, Yuhua Sun, Qianqian Zhang, Yang Liu, Jiaan Yang, Xiaojun Cai, Hong Qiao

**Affiliations:** 1 Department of Endocrinology, The Second Affiliated Hospital of Harbin Medical University, Harbin, Heilongjiang, China; 2 Heilongjiang Academy of Chinese Medical Sciences, Harbin, China; 3 Heilongjiang Provincial Maternal and Child Health Hospital, Harbin, China; 4 e-Health Research Institute, School of Management, Harbin Institute of Technology, Harbin, China; 5 The Second Affiliated Hospital of Harbin Medical University, Harbin, Heilongjiang, China

**Keywords:** adolescents, electronic-device use, indirect effect, overweight, physical activity

## Abstract

**Introduction:**

Childhood overweight and obesity are major public health concerns in China. The activity displacement hypothesis provides a plausible framework linking electronic-device use, physical activity, and weight status, but its applicability may vary across settings. This study examined overweight/obesity and explored whether physical-activity frequency formed a statistical pathway between daily electronic-device use and overweight/obesity among school-aged children and adolescents in Heilongjiang Province, China.

**Methods:**

This cross-sectional secondary analysis used data collected between October 2024 and April 2025 from participating schools in Daqing, Heihe, and Yichun/Wangkui. Of 26,535 survey records, 14,095 had nonmissing values for the outcome, exposure, age, and prespecified covariates and were retained in the original analysis. The questionnaire recorded average total daily electronic-device use, exercise frequency, nighttime sleep duration, and parental education. Multivariable logistic regression and sex- and grade-stratified models were fitted. To harmonize exposure specification, additional logistic models treated daily electronic-device use continuously, with and without adjustment for physical-activity frequency; school-clustered robust standard errors were examined as a sensitivity analysis. An exploratory bootstrap decomposition with 1,000 iterations estimated direct and indirect statistical associations through physical-activity frequency.

**Results:**

Among 14,095 participants, the prevalence of overweight/obesity was 38.2%. Male sex showed the strongest association (OR = 2.67, 95% CI: 2.48–2.87). A higher physical-activity frequency score (OR = 0.94) and a higher approximate sleep-duration score (OR = 0.96) were inversely associated with overweight/obesity. In the added continuous-exposure analysis, each additional reported hour/day was weakly associated before adjustment for physical activity (OR = 1.019, 95% CI: 1.002–1.035, *p* = 0.025) but not after adjustment (OR = 1.014, 95% CI: 0.998–1.031, *p* = 0.085); neither estimate was statistically significant with school-clustered robust standard errors. The exploratory decomposition yielded a direct probability difference of 0.0033 and an indirect probability difference of 0.0011 through physical-activity frequency; the descriptive indirect proportion was 24.35%.

**Conclusion:**

The association between reported daily electronic-device use and overweight/obesity was small and sensitive to exposure specification, physical-activity adjustment, and variance estimation. The fitted models were compatible with a small indirect statistical association through physical-activity frequency, but the cross-sectional data do not establish temporal ordering or causal mediation. Longitudinal studies using validated measures are needed.

## 1 Introduction

The global prevalence of overweight and obesity among children and adolescents has increased markedly over the past four decades, emerging as one of the most pressing public health challenges of the twenty-first century (44). According to the World Health Organization, the number of children and adolescents aged 5–19 years living with obesity increased more than tenfold between 1975 and 2016, rising from 11 million to 124 million worldwide (1). In low- and middle-income countries, this epidemic has accelerated alongside rapid economic development, urbanization, and shifts in dietary patterns and physical activity levels (2). China, as the world’s most populous nation and one of the fastest-growing economies, has experienced a particularly dramatic increase in childhood obesity prevalence. National surveillance data indicate that the combined prevalence of overweight and obesity among Chinese school-aged children and adolescents reached approximately 19.0% in 2014 and has continued to rise in subsequent years (3–5). The health consequences of childhood obesity extend well beyond the pediatric period: obese children are at substantially elevated risk of developing type 2 diabetes, hypertension, dyslipidemia, non-alcoholic fatty liver disease, and psychosocial disorders, and obesity in childhood strongly predicts obesity and cardiometabolic disease in adulthood (6–8, 45).

The aetiology of childhood obesity is multifactorial, involving genetic, dietary, behavioral, family, and socioeconomic influences (9). Screen-based behavior includes television viewing and the use of smartphones, tablets, computers, and gaming devices. Different types and purposes of device use may have different behavioral correlates. Prolonged screen exposure has been associated with overweight and obesity in systematic reviews and meta-analyses (10–14), although associations vary by measurement method and population.

The activity displacement hypothesis proposes that time spent in screen-based sedentary behavior may replace opportunities for physical activity (15). Other possible pathways include food-advertising exposure, eating during device use, and sleep disruption (16–19). Recent studies of early adolescents have considered screen exposure, physical activity, nutritional behaviors, weight status, and psychosocial well-being as interrelated domains rather than isolated risk factors (20, 21). This broader perspective is important because a statistical indirect effect through activity does not exclude dietary, sleep-related, or contextual pathways.

Physical activity is associated with multiple health benefits in children and adolescents (22, 23), and screen-based sedentary behavior and physical activity may compete for time (24). The specific gap addressed here is not whether displacement is possible, but whether the observed associations in a large cold-region school survey are compatible with an activity-related statistical pathway after adjustment for available covariates. In cross-sectional data, such a pathway is hypothesis-generating and cannot by itself establish temporal mediation.

Heilongjiang Province provides a relevant context because long, cold winters may constrain outdoor activity and the burden of childhood obesity is high in northeastern China (25). The study contributes a large multicenter description from participating school populations and an exploratory decomposition of the electronic-device use, physical-activity frequency, and overweight/obesity associations. The contribution is therefore contextual and empirical rather than evidence of a new causal mechanism.

The objectives were to: (1) describe overweight/obesity prevalence in the original analytic sample; (2) estimate associations with demographic and behavioral factors; (3) describe sex- and grade-specific patterns; and (4) explore, without causal interpretation, whether physical-activity frequency formed an indirect statistical pathway between reported daily electronic-device use and overweight/obesity.

## 2 Methods

### 2.1 Study design and setting

This cross-sectional secondary analysis used school survey data collected between October 2024 and April 2025 from participating schools in three study regions of Heilongjiang Province: Daqing, Heihe, and Yichun/Wangkui. These sites included urban, suburban, and rural school settings. The database contained 26,535 survey records, but the number of students originally invited and the number who returned no questionnaire were not available. Accordingly, 26,535 is described as the number of available records, not the invitation denominator or a survey response rate. Because sampling probabilities for all schools in the province were unavailable and 73.3% of the analytic sample came from Daqing, the results are interpreted primarily for the participating schools, especially those in Daqing, rather than as a province-wide probability sample.

### 2.2 Sampling strategy and participants

The survey used a multistage school-based approach in which participating schools and classes contributed students present during data collection. This manuscript is a secondary analysis of an existing survey; no analysis-specific *a priori* sample-size calculation governed recruitment. We therefore describe how the analytic study size was obtained and provide a *post hoc* precision benchmark, not a retrospective sampling justification. All 26,535 available survey records were screened, and the 14,095 records meeting the stated complete-case eligibility criteria formed the analytic sample. Using the standard large-sample precision formula for a proportion, *d* = Z sqrt[DEFF x p(1-p)/n] (26, 27), *n* = 14,095 and *Z* = 1.96 yield a worst-case 95% half-width of 0.83 percentage points under simple random sampling because *p* = 0.50 maximizes p(1-p) (48, 49). In an illustrative sensitivity scenario in which clustering doubles the variance (DEFF = 2.0), the half-width is 1.17 percentage points. DEFF = 2.0 was not estimated from the survey and is not presented as an original design parameter. No nonresponse inflation is applied because this benchmark uses the final analytic sample. It addresses descriptive prevalence precision only and does not remove possible selection bias or establish power for every regression or indirect-effect estimate.

Records were eligible for the original analysis when the overweight/obesity outcome, daily electronic-device use, age, sex, grade group, nighttime sleep-duration score, physical-activity frequency score, parental-education score, and geographic region were nonmissing. The original analysis used a complete-case cohort; the implications of this choice are evaluated in a comparison of included and excluded records and in the limitations.

A total of 26,535 survey records were available. Sequential exclusions for unavailable or implausible height/weight information, incomplete core covariates, missing electronic-device use, and missing age produced the original analytic sample of 14,095. Figure 1 shows the corresponding record-level flow without changing the original sample size or primary estimates. Because the database did not contain the total number invited or students who returned no questionnaire, this flow quantifies analytic exclusion among available records rather than overall survey nonresponse.

**Figure 1:**
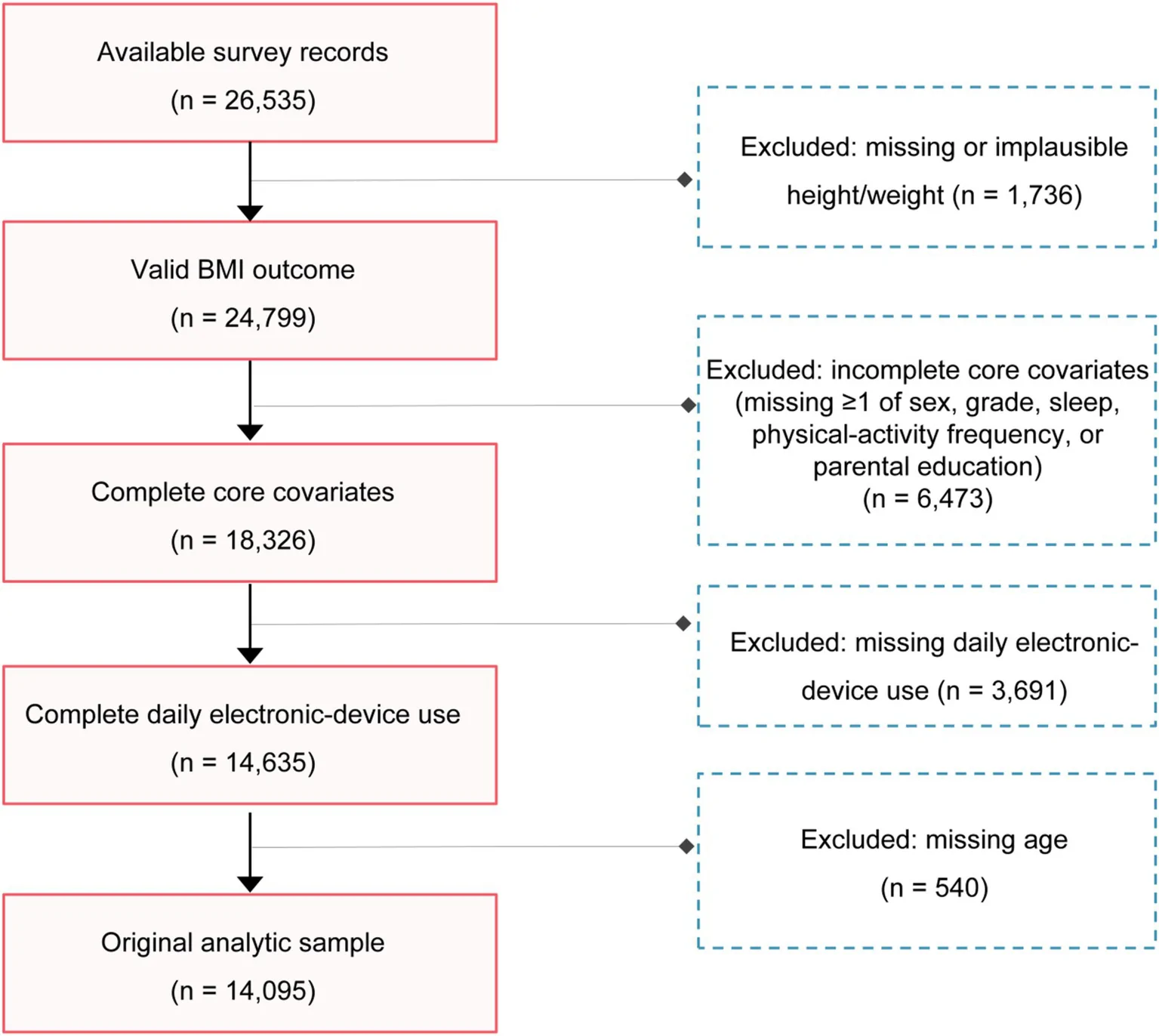
Participant flow for the original analytic sample.

Of 26,535 records, 24,799 had a valid BMI outcome, 18,326 had complete core covariates, 14,635 also had electronic-device-use data, and 14,095 had nonmissing age. The 6,473 incomplete core-covariate records could be missing one or more of sex, grade, sleep, physical-activity frequency, or parental education. The final original analytic sample remained 14,095.

### 2.3 Anthropometric measurements

The questionnaire contained fields for height in centimeters and weight in kilograms. The available materials did not document whether these values were directly measured by school staff or reported by participants or parents, nor did they contain replicate readings or device-calibration records. BMI was calculated as weight in kilograms divided by height in meters squared (kg/m2). Missing or implausible height or weight values were excluded. Measurement error cannot be ruled out.

Overweight and obesity were defined according to the Chinese national standard for screening overweight and obesity in school-aged children and adolescents (WS/T 586—2018), which provides age- and sex-specific BMI cut-off values for children aged 6–18 years. This standard is widely used in Chinese epidemiological research and surveillance and is recommended by the National Health Commission of China. The binary outcome variable was coded as 1 (overweight or obese) and 0 (non-overweight/obese).

### 2.4 Assessment of screen time

Participants reported their average total time using electronic devices per day in hours and minutes. The item referred broadly to devices such as smartphones, tablets, smart watches, and computers. The printed questionnaire states that participating students complete the survey and places this item in the student-facing section; a later section is separately labelled for parent completion. However, the analytic dataset did not record who entered each response or whether younger children received assistance from a parent or teacher. The item did not distinguish academic from recreational use or weekdays from weekends and had no documented psychometric validation. The variable is therefore described as self-reported daily electronic-device use. It was analyzed categorically as no use, <=2 h/day, 2–4 h/day, and >4 h/day in the original logistic model and continuously in the added harmonized logistic models and exploratory decomposition.

### 2.5 Assessment of physical activity

Physical-activity frequency was assessed in the student-facing section with the question, ‘What is your average frequency of exercise (including physical education classes)?’ Response options were never, several times per year or less, several times per month, several times per week, and daily. For the original analysis, these responses were converted to an approximate weekly-frequency score (0, 0.5, 1, 3, and 7, respectively). This study-specific single item had no documented reliability or validity assessment. The variable does not measure days meeting a 30- or 60-min moderate-to-vigorous physical-activity guideline and should not be interpreted as guideline compliance.

### 2.6 Assessment of other variables

Nighttime sleep duration during the previous month was reported in five categories: <=6 h, 6–7 h, 7–8 h, 8–9 h, and >9 h. The original analysis represented these ordered responses with approximate hour values. The resulting coefficient is interpreted as an ordinal/approximate trend rather than a precisely measured one-hour causal effect.

Maternal and paternal education were each reported in five categories: junior high school or below, senior high/vocational school, junior college, bachelor’s degree, and master’s degree or above. The higher reported parental level was coded from 1 to 5. The original regression treated this as an ordinal trend variable; this assumes a constant log-odds change between adjacent categories and is interpreted cautiously rather than as a psychometric scale. The exact questionnaire wording, intended respondent, original analytical coding, and measurement limitations are summarized in Supplementary Table S1.

Myopia status was self-reported by participants or their parents as a binary variable (yes/no).

Geographic region was categorized into three strata based on study site: Daqing and surrounding areas (reference category), Heihe, and Yichun/Wangkui.

Sex was recorded as male or female, and grade level was categorized into four groups: lower primary school (Grades 1–3), upper primary school (Grades 4–6), junior high school (Grades 7–9), and senior high school (Grades 10–12).

### 2.7 Statistical analysis

Descriptive statistics were used to characterize the study population overall and by overweight/obesity status. Continuous variables are presented as means ± standard deviations (SD) or medians with interquartile ranges (IQR) as appropriate; categorical variables are presented as frequencies and percentages. Between-group differences were assessed using independent-samples *t*-tests or Mann–Whitney *U* tests for continuous variables and Pearson chi-squared tests for categorical variables.

Multivariable binary logistic regression was used to examine factors associated with overweight/obesity. Results are expressed as odds ratios (ORs) with 95% confidence intervals (CIs). The original models categorized daily electronic-device use as no use, <=2 h/day (reference), 2–4 h/day, and >4 h/day; physical activity, approximate sleep duration, and parental education were entered as ordinal scores. In response to the review, two harmonized continuous-exposure models estimated the OR per additional reported hour/day: a total-association model without physical-activity frequency and a model additionally adjusted for the original physical-activity frequency score. Conventional standard errors were reported for comparability with the submitted model, and school-clustered robust standard errors were added as a sensitivity analysis. The categorical screen-use terms were also tested jointly.

Sex- and grade-stratified models were retained as descriptive subgroup analyses. Because formal interaction tests were not part of the original analysis, differences between subgroup-specific significance levels are not interpreted as proof of effect modification.

An exploratory model-based decomposition was performed using the mediation package (version 4.5.0) in R. The mediator model regressed the physical-activity frequency score on continuous daily electronic-device use and covariates. The outcome model was a logistic regression of overweight/obesity on continuous device use, physical-activity frequency, and the same covariates. The software-reported ACME, ADE, total effect, and proportion mediated were estimated with 1,000 nonparametric bootstrap resamples. In this manuscript, ACME and ADE are retained only as software output labels; the estimates are described as indirect and direct probability differences without causal interpretation.

The decomposition requires strong assumptions about temporal ordering and unmeasured confounding that cannot be verified in this cross-sectional survey. Exposure, physical activity, and weight status were measured at the same time, and reverse causation is possible. Diet, family lifestyle, and other sedentary behaviors may confound the fitted pathways. The indirect estimate is therefore interpreted as an exploratory statistical association compatible with, but not proof of, the activity displacement hypothesis.

The original analyses used R software (version 4.3.x). The added sensitivity models were estimated in Python 3.12 by maximum-likelihood logistic regression; the implementation was validated by reproducing the submitted Table 1 estimates, and sandwich covariance estimates accounted for clustering by school. Two-sided *p* < 0.05 was used. The primary results retain the original complete-case analysis. Characteristics of the 14,095 included and 12,440 excluded records were compared using available data (Supplementary Table S2), and record-retention proportions were calculated within available grade and region categories (Supplementary Table S4). Only 2,979 excluded records had available device-use time, and observed differences in age, grade, region, and behaviors indicate that missingness should not be assumed completely random. Multiple imputation was not undertaken because most excluded records lacked the exposure and the available auxiliary information was insufficient to support reliable extrapolation; selection bias remains possible. The mean physical-activity score difference and its 95% confidence interval were calculated from the group means, standard deviations, and sample sizes.

**Table 1:** Multivariable logistic regression analysis of factors associated with overweight/obesity among school-aged children and adolescents.

Variable	Model 1 OR (95%CI)	Model 1 *p*-value	Model 2 OR (95%CI)	Model 2 *p*-value
Sex (male vs. female)	2.67 (2.49–2.87)	<0.001	2.67 (2.48–2.87)	<0.001
Grade level (ref: senior high school)				
Junior high school	1.31 (1.19–1.44)	<0.001	1.32 (1.20–1.47)	<0.001
Upper primary school	1.57 (1.40–1.77)	<0.001	1.59 (1.41–1.80)	<0.001
Lower primary school	1.42 (1.07–1.88)	0.013	1.44 (1.08–1.90)	0.011
Approximate sleep-duration score (hours)	0.96 (0.93–1.00)	0.027	0.96 (0.93–1.00)	0.029
Physical-activity frequency score	0.94 (0.92–0.95)	<0.001	0.94 (0.92–0.95)	<0.001
Highest parental-education ordinal score	0.96 (0.93–0.98)	0.003	0.96 (0.93–0.99)	0.004
Daily electronic-device use (ref: ≤2 h/day)				
No screen use	—	—	0.99 (0.79–1.23)	0.922
2–4 h/day	—	—	1.03 (0.93–1.14)	0.536
>4 h/day	—	—	1.04 (0.93–1.18)	0.479
Geographic region (ref: Daqing)				
Heihe	1.71 (1.33–2.20)	<0.001	1.70 (1.32–2.19)	<0.001
Yichun/Wangkui	0.94 (0.86–1.03)	0.21	0.94 (0.85–1.03)	0.165

Model 1 adjusted for sex, grade level, approximate sleep-duration score, physical-activity frequency score, highest parental-education ordinal score, and geographic region. Model 2 additionally included categorical daily electronic-device use. OR, odds ratio; CI, confidence interval.

## 3 Results

### 3.1 Characteristics of study participants

A total of 14,095 participants with complete data were included in the final analysis, comprising 6,947 males (49.3%) and 7,148 females (50.7%). The median age of the overall sample was 13.00 years (IQR: 12.00–14.00). By grade level, 1.7% of participants were in lower primary school (Grades 1–3), 21.9% in upper primary school (Grades 4–6), 51.1% in junior high school (Grades 7–9), and 25.4% in senior high school (Grades 10–12). Geographically, 73.3% of participants were from Daqing and surrounding areas, 24.7% from Yichun/Wangkui, and 2.0% from Heihe.

The overall prevalence of overweight/obesity was 38.2% (*n* = 5,390). Table 2 presents the baseline characteristics of participants stratified by overweight/obesity status. Statistically significant differences between the overweight/obese and non-overweight/obese groups were observed for sex, grade level, age, BMI, physical activity frequency, screen time, myopia status, and geographic region (all *p* < 0.05). In contrast, no statistically significant differences were found for sleep duration (*p* = 0.203) or parental education level (*p* = 0.326) in univariable comparisons.

**Table 2:** Baseline characteristics of study participants by overweight/obesity status (*n* = 14,095).

**Variable**	**Category**	**Total (*n* = 14,095)**	**Non-overweight/obese (*n* = 8,705)**	**Overweight/obese (*n* = 5,390)**	***p*-value**
Sex	Male	6,947 (49.3%)	3,540 (40.7%)	3,407 (63.2%)	<0.001
	Female	7,148 (50.7%)	5,165 (59.3%)	1,983 (36.8%)	
Grade level	Senior high school	3,574 (25.4%)	2,324 (26.7%)	1,250 (23.2%)	<0.001
	Junior high school	7,198 (51.1%)	4,449 (51.1%)	2,749 (51.0%)	
	Upper primary school	3,081 (21.9%)	1,788 (20.5%)	1,293 (24.0%)	
	Lower primary school	242 (1.7%)	144 (1.7%)	98 (1.8%)	
Age (years)	Median [IQR]	13.00 [12.00, 14.00]	13.00 [12.00, 15.00]	13.00 [11.00, 14.00]	<0.001
BMI (kg/m^2^)	Median [IQR]	20.32 [17.69, 24.22]	18.31 [16.66, 19.94]	25.54 [23.53, 28.68]	<0.001
Approximate sleep-duration score (hours)	Mean ± SD	7.15 ± 1.10	7.15 ± 1.09	7.17 ± 1.11	0.203
Physical-activity frequency score	Mean ± SD	3.30 ± 2.37	3.37 ± 2.40	3.20 ± 2.33	<0.001
Daily electronic-device use (hours/day)	Median [IQR]	1.17 [0.50, 2.50]	1.08 [0.50, 2.50]	1.20 [0.50, 2.50]	0.039
Myopia	No	3,547 (25.8%)	2,076 (24.4%)	1,471 (28.1%)	<0.001
	Yes	10,194 (74.2%)	6,427 (75.6%)	3,767 (71.9%)	
Highest parental-education ordinal score	Mean ± SD	2.78 ± 1.32	2.79 ± 1.32	2.77 ± 1.31	0.326
Geographic region	Daqing	10,332 (73.3%)	6,356 (73.0%)	3,976 (73.8%)	<0.001
	Heihe	279 (2.0%)	129 (1.5%)	150 (2.8%)	
	Yichun/Wangkui	3,484 (24.7%)	2,220 (25.5%)	1,264 (23.5%)	

BMI, body mass index; IQR, interquartile range; SD, standard deviation.

Physical-activity frequency score: never = 0, several times/year or less = 0.5, several times/month = 1, several times/week = 3, and daily = 7. Highest parental-education ordinal score: 1 = junior high school or below; 2 = senior high/vocational school; 3 = junior college; 4 = bachelor’s degree; 5 = master’s degree or above. For the physical-activity score, the overweight/obesity minus non-overweight/obesity mean difference was −0.17 points (95% CI: −0.25 to −0.09).

The sex distribution differed markedly between groups: among participants with overweight/obesity, 63.2% were male, compared with 40.7% among those without overweight/obesity (*p* < 0.001). Median BMI was 25.54 kg/m2 (IQR: 23.53–28.68) in the overweight/obese group and 18.31 kg/m2 (IQR: 16.66–19.94) in the non-overweight/obese group. The physical-activity frequency score was lower in the overweight/obese group (3.20 +/− 2.33) than in the non-overweight/obese group (3.37 +/− 2.40), but the absolute difference was small: −0.17 points on the constructed 0–7 score (95% CI: −0.25 to −0.09; *p* < 0.001). The approximate sleep-duration score was similar between groups (7.17 +/− 1.11 vs. 7.15 +/− 1.09; *p* = 0.203). Median daily electronic-device use was 1.20 h (IQR: 0.50–2.50) and 1.08 h (IQR: 0.50–2.50), respectively (*p* = 0.039). The former Figure 2 is retained as Supplementary Figure S1.

Among records with grade available, 242 of 425 lower-primary records (56.9%), 3,081 of 9,102 upper-primary records (33.8%), 7,198 of 11,265 junior-high records (63.9%), and 3,574 of 5,416 senior-high records (66.0%) were retained. These proportions describe retention among records already present in the database; they are not questionnaire response rates because the invitation denominator was unavailable.

By region, 10,332 of 18,159 available Daqing records (56.9%), 3,484 of 7,560 Yichun/Wangkui records (46.1%), and 279 of 816 Heihe records (34.2%) were retained. The analytic sample was therefore concentrated in Daqing (73.3%), and regional imbalance limits province-wide generalization (Supplementary Table S4).

### 3.2 Factors associated with overweight/obesity: multivariable logistic regression

Two multivariable logistic regression models were constructed: Model 1 excluded screen time, and Model 2 included screen time as an additional exposure variable. Results of both models are presented in Table 1. The two models yielded highly consistent results across all covariates, indicating that the inclusion of screen time did not materially alter the associations of other variables with overweight/obesity.

Sex was the strongest independent predictor of overweight/obesity in both models. In Model 2, male students had 2.67 times the odds of overweight/obesity compared with female students (OR = 2.67, 95%CI: 2.48–2.87, *p* < 0.001).

Grade level was significantly associated with overweight/obesity risk. Using senior high school students as the reference group, upper primary school students had the highest odds of overweight/obesity (OR = 1.59, 95%CI: 1.41–1.80, *p* < 0.001), followed by lower primary school students (OR = 1.44, 95%CI: 1.08–1.90, *p* = 0.011) and junior high school students (OR = 1.32, 95%CI: 1.20–1.47, *p* < 0.001). This pattern indicates a consistent inverse relationship between grade level and overweight/obesity risk.

The physical-activity frequency score was inversely associated with overweight/obesity (OR = 0.94, 95% CI: 0.92–0.95, *p* < 0.001) per one-point increase in the original approximate weekly-frequency coding. This corresponds to a modest 6% lower odds per score point and should not be described as a large protective effect or as evidence of clinical importance. The approximate sleep-duration score was also inversely associated (OR = 0.96, 95% CI: 0.93–1.00, *p* = 0.029). These coefficients are trends across constructed scores and should not be interpreted as effects of meeting movement or sleep guidelines.

The highest parental-education ordinal score was inversely associated with overweight/obesity after covariate adjustment (OR = 0.96, 95% CI: 0.93–0.99, *p* = 0.004). Because the model assumes a constant log-odds change between adjacent education categories, the estimate is interpreted as an exploratory ordinal trend.

Geographic region was significantly associated with overweight/obesity risk. Compared with Daqing, participants from Heihe had significantly higher odds of overweight/obesity (OR = 1.70, 95%CI: 1.32–2.19, *p* < 0.001), while those from Yichun/Wangkui did not differ significantly from the reference (OR = 0.94, 95%CI: 0.85–1.03, *p* = 0.165).

Categorical daily electronic-device use was not statistically associated with overweight/obesity after adjustment: no use (OR = 0.99, 95% CI: 0.79–1.23, *p* = 0.922), 2–4 h/day (OR = 1.03, 95% CI: 0.93–1.14, *p* = 0.536), and >4 h/day (OR = 1.04, 95% CI: 0.93–1.18, *p* = 0.479), relative to <=2 h/day; the three terms were not jointly significant (Wald *p* = 0.866). In the added continuous-exposure analysis, the total-association model gave OR = 1.019 per additional hour/day (95% CI: 1.002–1.035, *p* = 0.025). After including the original physical-activity frequency score, the estimate attenuated to OR = 1.014 (95% CI: 0.998–1.031, *p* = 0.085). With school-clustered robust standard errors, the corresponding *p*-values were 0.069 and 0.156 (Supplementary Table S3). Thus, the device-use association is small and sensitive to adjustment and variance specification.

### 3.3 Subgroup analyses by sex

Results of the sex-stratified models are retained in Table 3; the former Figure 3 is provided as Supplementary Figure S2. The physical-activity frequency score was inversely associated with overweight/obesity in males (OR = 0.93, 95% CI: 0.91–0.94, *p* < 0.001) and females (OR = 0.96, 95% CI: 0.94–0.98, *p* < 0.001). Because no formal interaction test was conducted in the original analysis, the numerical difference is described rather than interpreted as established effect modification.

**Table 3:** Sex-stratified multivariable logistic regression analysis of factors associated with overweight/obesity.

Variable	Male (*n* = 6,947) OR (95%CI)	Male *p*-value	Female (*n* = 7,148) OR (95%CI)	Female *p*-value
Grade level (ref: senior high school)				
Junior high school	1.42 (1.24–1.63)	<0.001	1.20 (1.03–1.40)	0.018
Upper primary school	1.70 (1.44–2.01)	<0.001	1.45 (1.21–1.75)	<0.001
Lower primary school	1.50 (1.03–2.19)	0.033	1.32 (0.86–2.00)	0.198
Approximate sleep-duration score (hours)	0.96 (0.92–1.00)	0.078	0.96 (0.91–1.01)	0.159
Physical-activity frequency score	0.93 (0.91–0.94)	<0.001	0.96 (0.94–0.98)	<0.001
Highest parental-education ordinal score	0.99 (0.95–1.03)	0.588	0.92 (0.88–0.96)	<0.001
Daily electronic-device use (ref: ≤2 h/day)				
No screen use	0.83 (0.62–1.12)	0.223	1.23 (0.88–1.68)	0.213
2–4 h/day	1.07 (0.93–1.23)	0.347	1.00 (0.85–1.16)	0.951
>4 h/day	1.01 (0.86–1.19)	0.901	1.09 (0.91–1.31)	0.34
Geographic region (ref: Daqing)				
Heihe	1.79 (1.23–2.64)	0.003	1.67 (1.18–2.34)	0.003
Yichun/Wangkui	0.92 (0.81–1.04)	0.194	0.95 (0.83–1.10)	0.507

The highest parental-education ordinal score was inversely associated among girls (OR = 0.92, 95% CI: 0.88–0.96, *p* < 0.001) but not among boys (OR = 0.99, 95% CI: 0.95–1.03, *p* = 0.588). This contrast is exploratory because the models assume a linear ordinal trend and no formal sex-by-education interaction test was reported. The approximate sleep-duration score was not statistically significant in either sex subgroup.

Categorical daily electronic-device use was not statistically associated with overweight/obesity in either sex subgroup (all *p* > 0.05), consistent with the categorical overall model. Geographic region showed similar patterns across sexes: compared with Daqing, Heihe was associated with higher odds among both males and females.

### 3.4 Subgroup analyses by grade level

Results of grade-stratified multivariable logistic regression analyses are presented in Table 4.

**Table 4:** Grade-stratified multivariable logistic regression analysis of factors associated with overweight/obesity.

Grade level	Variable	OR (95%CI)	*p*-value
Lower primary school (*n* = 242)	Sex (male vs. female)	2.94 (1.71–5.16)	<0.001
	Approximate sleep-duration score	0.93 (0.71–1.20)	0.571
	Physical-activity frequency score	0.96 (0.85–1.09)	0.549
	Highest parental-education ordinal score	0.84 (0.68–1.05)	0.123
	Daily electronic-device use: no use	—	—
	Daily electronic-device use: 2–4 h/day	0.56 (0.24–1.23)	0.157
	Daily electronic-device use: >4 h/day	1.03 (0.36–2.90)	0.962
	Region: Heihe	—	—
	Region: Yichun/Wangkui	0.38 (0.08–1.44)	0.181
Upper primary school (*n* = 3,081)	Sex (male vs. female)	2.87 (2.47–3.35)	<0.001
	Approximate sleep-duration score	0.90 (0.84–0.97)	0.004
	Physical-activity frequency score	0.93 (0.90–0.96)	<0.001
	Highest parental-education ordinal score	0.93 (0.88–0.99)	0.018
	Daily electronic-device use: no use	0.90 (0.58–1.38)	0.633
	Daily electronic-device use: 2–4 h/day	1.01 (0.79–1.29)	0.938
	Daily electronic-device use: >4 h/day	1.04 (0.69–1.55)	0.868
	Region: Heihe	2.80 (1.73–4.65)	<0.001
	Region: Yichun/Wangkui	1.68 (1.27–2.22)	<0.001
Junior high school (*n* = 7,198)	Sex (male vs. female)	2.83 (2.56–3.13)	<0.001
	Approximate sleep-duration score	0.97 (0.93–1.02)	0.269
	Physical-activity frequency score	0.93 (0.91–0.95)	<0.001
	Highest parental-education ordinal score	0.95 (0.92–0.99)	0.022
	Daily electronic-device use: no use	1.03 (0.77–1.38)	0.819
	Daily electronic-device use: 2–4 h/day	1.05 (0.91–1.21)	0.51
	Daily electronic-device use: >4 h/day	1.08 (0.87–1.34)	0.466
	Region: Heihe	1.09 (0.72–1.63)	0.682
	Region: Yichun/Wangkui	0.92 (0.80–1.05)	0.196
Senior high school (*n* = 3,574)	Sex (male vs. female)	2.16 (1.87–2.50)	<0.001
	Approximate sleep-duration score	1.01 (0.93–1.09)	0.826
	Physical-activity frequency score	0.96 (0.93–0.99)	0.019
	Highest parental-education ordinal score	1.02 (0.95–1.08)	0.623
	Daily electronic-device use: no use	1.04 (0.58–1.81)	0.891
	Daily electronic-device use: 2–4 h/day	1.05 (0.87–1.27)	0.598
	Daily electronic-device use: >4 h/day	1.08 (0.91–1.28)	0.362
	Region: Heihe	1.50 (0.93–2.39)	0.092
	Region: Yichun/Wangkui	0.82 (0.71–0.96)	0.012

Reference categories: sex = female; daily electronic-device use = <=2 h/day; geographic region = Daqing. A dash indicates that the variable was not estimable because of insufficient sample size in that stratum. OR, odds ratio; CI, confidence interval.

Sex was a significant predictor of overweight/obesity across all grade groups, with the OR for male sex showing a declining trend with advancing grade level: OR = 2.94 (95%CI: 1.71–5.16) in lower primary school, OR = 2.87 (95%CI: 2.47–3.35) in upper primary school, OR = 2.83 (95%CI: 2.56–3.13) in junior high school, and OR = 2.16 (95%CI: 1.87–2.50) in senior high school. This progressive attenuation suggests that the sex disparity in overweight/obesity risk narrows as children advance through the school system.

The physical-activity frequency score was inversely associated in upper primary school (OR = 0.93, 95% CI: 0.90–0.96, *p* < 0.001), junior high school (OR = 0.93, 95% CI: 0.91–0.95, *p* < 0.001), and senior high school (OR = 0.96, 95% CI: 0.93–0.99, *p* = 0.019), but not in lower primary school (OR = 0.96, 95% CI: 0.85–1.09, *p* = 0.549). These stratum-specific estimates are descriptive and may be imprecise in the smallest stratum (*n* = 242).

Sleep duration was a significant protective factor only in the upper primary school group (OR = 0.90, 95%CI: 0.84–0.97, *p* = 0.004), with non-significant associations in all other grade groups (lower primary: *p* = 0.571; junior high: *p* = 0.269; senior high: *p* = 0.826).

The parental-education ordinal trend was statistically significant in upper primary school (OR = 0.93, 95% CI: 0.88–0.99, *p* = 0.018) and junior high school (OR = 0.95, 95% CI: 0.92–0.99, *p* = 0.022), but not in lower primary or senior high school. These exploratory results should not be interpreted as proof of grade-specific effect modification.

Categorical daily electronic-device use was not statistically associated with overweight/obesity in any grade stratum (all *p* > 0.05). This is consistent with the categorical overall model but does not resolve the difference from the continuous exposure specification used in the decomposition.

Geographic region showed heterogeneous patterns across grade levels. The elevated risk associated with Heihe was most pronounced in upper primary school students (OR = 2.80, 95%CI: 1.73–4.65, *p* < 0.001) but was not statistically significant in junior high school (*p* = 0.682) or senior high school (*p* = 0.092) students. In the Yichun/Wangkui stratum, a significantly lower risk was observed in senior high school students (OR = 0.82, 95%CI: 0.71–0.96, *p* = 0.012) and a significantly higher risk in upper primary school students (OR = 1.68, 95%CI: 1.27–2.22, *p* < 0.001) compared with Daqing.

### 3.5 Exploratory indirect-effect decomposition

Using the physical-activity frequency score as the intermediate variable, the original 1,000-resample bootstrap analysis estimated a total probability difference of 0.0044 (95% CI: 0.0011–0.0080, *p* = 0.012), a direct probability difference of 0.0033 (95% CI: 0.0001–0.0070, *p* = 0.044), and an indirect probability difference of 0.0011 (95% CI: 0.0007–0.0014, *p* < 0.001). The descriptive indirect proportion was 24.35% (95% CI: 10.93–85.53%, *p* = 0.012). These numerical estimates are unchanged from the original analysis. They describe a fitted cross-sectional statistical pathway and do not establish that electronic-device use preceded lower activity or higher body weight (Table 5 and Figure 2).

**Table 5:** Exploratory bootstrap decomposition of the association between daily electronic-device use and overweight/obesity through physical-activity frequency (*n* = 14,095).

**Effect**	**Estimate**	**95%CI**	**p-value**
Total effect	0.0044	0.0011–0.0080	0.012
Estimated direct effect (software ADE)	0.0033	0.0001–0.0070	0.044
Estimated indirect effect (software ACME)	0.0011	0.0007–0.0014	<0.001
Proportion mediated	0.2435	10.93–85.53%	0.012
a-path: daily electronic-device use → physical-activity frequency score (β)	−0.075	—	<0.001
b-path: physical-activity frequency score → overweight/obesity (β)	−0.064	—	<0.001

Effects are probability differences. ADE and ACME are retained in this table only to identify the software output; they are interpreted here as estimated direct and indirect statistical associations. Bootstrap confidence intervals used 1,000 nonparametric resamples. Cross-sectional temporal ordering and no-unmeasured-confounding assumptions cannot be verified.

**Figure 2:**
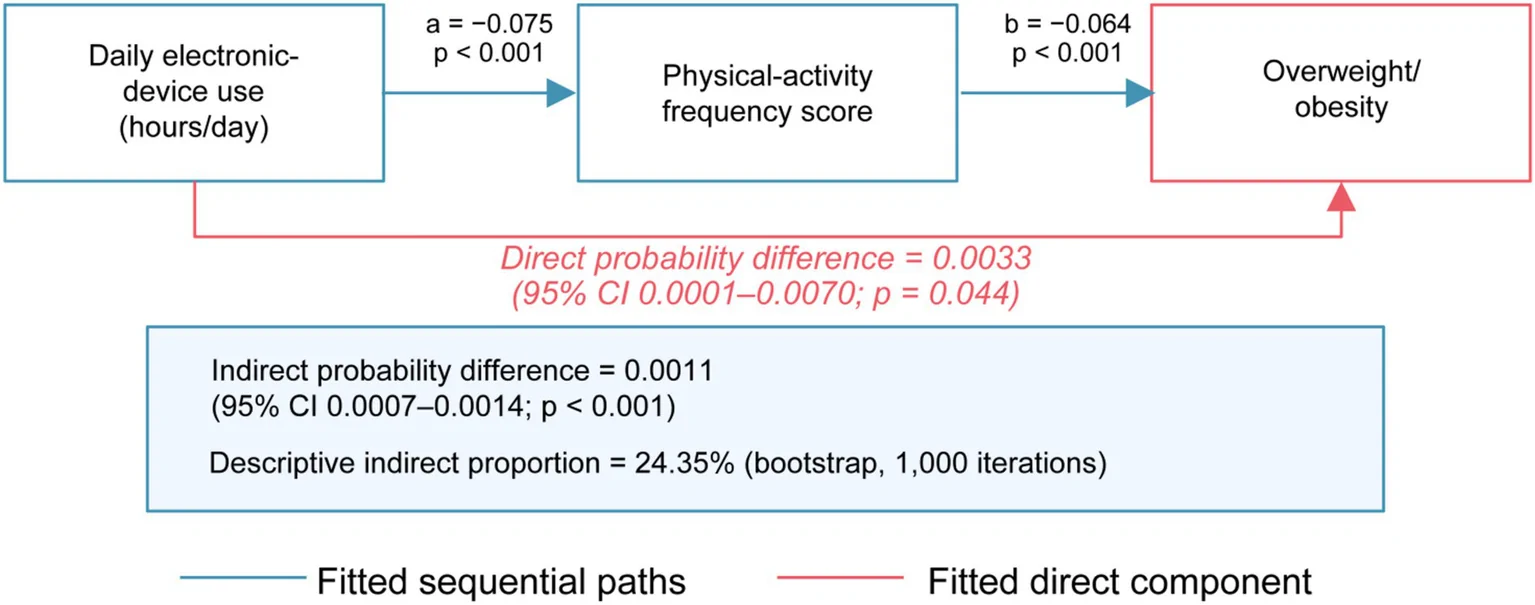
Exploratory statistical pathway through physical-activity frequency in the original analytic sample (*n* = 14,095).

## 4 Discussion

### 4.1 Principal findings

In this cross-sectional study of 14,095 school-aged participants, overweight/obesity prevalence was 38.2%. Male sex, younger grade level, a lower physical-activity frequency score, and a lower approximate sleep-duration score were associated with higher odds. The physical-activity association was statistically precise but modest in magnitude, and no clinically important difference was prespecified for the constructed score. The added harmonized continuous-exposure models showed a weak total association that was attenuated after physical-activity adjustment and was not statistically significant with school-clustered robust standard errors. The original decomposition estimated that the indirect component through physical-activity frequency represented 24.35% of the fitted total association, but the estimates are interpreted as exploratory cross-sectional probability differences rather than causal effects.

### 4.2 Prevalence of overweight/obesity

The observed prevalence was higher than estimates from several national Chinese studies (28, 29). Differences in geography, age composition, sampling, outcome measurement, and complete-case selection may contribute. Because the participating schools were concentrated in three study regions and the height/weight measurement protocol was not documented in the available materials, 38.2% should be interpreted as the prevalence in the analyzed school sample rather than a province-wide surveillance estimate.

### 4.3 Sex differences in overweight/obesity risk

The substantially higher odds of overweight/obesity among boys (OR = 2.67) is consistent with the well-documented pattern of male-predominant childhood obesity in China and other East Asian countries (30). This sex disparity likely reflects a confluence of biological, behavioral, and sociocultural factors. From a biological perspective, boys and girls differ in fat distribution, hormonal profiles, and pubertal timing, all of which influence BMI trajectories during childhood and adolescence (31). From a behavioral perspective, boys in China tend to have higher energy intake relative to expenditure, are more likely to engage in sedentary gaming and screen-based activities, and may be less responsive to parental dietary guidance (32). From a sociocultural perspective, cultural norms in China have historically associated a larger body size in boys with health and prosperity, potentially reducing parental concern about excess weight in sons (33).

The attenuation of the sex effect with advancing grade level — from OR = 2.94 in lower primary school to OR = 2.16 in senior high school — is an important finding that has not been widely reported in the Chinese literature. This trend may reflect the onset of puberty and the associated hormonal changes that promote fat accumulation in girls during adolescence, thereby narrowing the sex gap in BMI (34). Additionally, older adolescent girls may become increasingly aware of societal body image norms and engage in more deliberate weight management behaviors, including dietary restriction and increased physical activity (35). Longitudinal studies tracking sex-specific BMI trajectories from childhood through adolescence would be valuable for confirming and extending this observation.

### 4.4 Grade-level patterns and developmental considerations

The inverse association between grade level and overweight/obesity risk, with primary school students at highest risk relative to senior high school students, may reflect several developmental and environmental mechanisms. Younger children have less autonomy over food choices and are more dependent on parental feeding practices, which in China are often characterized by overfeeding and pressure to eat, particularly in families with a single child (36). The school food environment also changes with grade level: primary schools in China are more likely to provide canteen meals with limited nutritional regulation, whereas older students may have greater access to and preference for lower-calorie food options (37, 38).

The physical-activity frequency score was not statistically associated with overweight/obesity in the lower-primary stratum. The small lower-primary stratum reflects the composition of the available records; the invitation denominator and questionnaire return rate by age were not recorded. Among records with grade available, 56.9% of lower-primary records were retained, compared with 33.8% of upper-primary, 63.9% of junior-high, and 66.0% of senior-high records (Supplementary Table S4). These record-level proportions do not show that lower-primary records were uniquely less likely to enter the analytic sample, but they cannot exclude differential survey participation before records entered the database. The small stratum and use of an approximate frequency score limit interpretation. The questionnaire did not measure minutes of moderate-to-vigorous activity or compliance with the 60-min youth guideline; future work should use validated multi-item instruments or objective monitoring.

### 4.5 Screen time and overweight/obesity

Reported daily electronic-device use was a central exposure. In the adjusted logistic regression, the categorical exposure groups were not independently associated with overweight/obesity. This result indicates no clear categorical contrast at the selected cut points; it does not establish absence of an association under every exposure specification.

The added continuous logistic models help distinguish exposure coding from the decomposition itself. The total-association model was weakly positive with conventional standard errors, whereas the activity-adjusted continuous model was not statistically significant; neither model was significant with school-clustered robust standard errors. The original decomposition yielded statistically significant total and direct probability differences, but those results use a different estimand and bootstrap procedure. Taken together, the evidence is model- and variance-specification dependent and does not establish a robust independent device-use effect.

The device-use item combined academic, social, and recreational purposes and did not distinguish weekdays from weekends. Device use may be related to weight through activity displacement, eating during use, food marketing, sleep disruption, and other factors. Studies integrating screen exposure, physical activity, nutrition, and psychosocial outcomes support treating these domains as interrelated (20, 21). The present analysis cannot determine which device-use purposes or mechanisms are responsible (46, 47).

### 4.6 Physical activity as an exploratory statistical pathway

The fitted a-path (device use to physical-activity frequency score: beta = −0.075, *p* < 0.001) and b-path (physical-activity frequency score to overweight/obesity: beta = −0.064, *p* < 0.001) were statistically significant. The indirect component represented 24.35% of the fitted total association, but both the between-group activity-score difference and the adjusted activity OR indicate a small association. These unchanged estimates are compatible with the activity displacement hypothesis, but the cross-sectional design does not confirm temporal sequence or clinical importance.

Physical activity was examined because it was the prespecified intermediate variable in the activity-displacement framework, not because it had the largest odds ratio. The analysis was not designed to rank all risk factors or to claim that physical activity is more important than sex, grade, region, diet, or family context. The original bootstrap decomposition produced a statistically significant direct probability difference, but the added activity-adjusted continuous logistic model was not statistically significant, particularly with school-clustered robust uncertainty. The direct and indirect components should therefore be regarded as exploratory and small. Diet, snacking during device use, sleep disruption, family context, geographic differences, and non-screen sedentary time may contribute, and residual confounding remains possible (41).

The findings support testing integrated approaches to device-use management and physical-activity promotion, but they do not establish that reducing device use will cause weight reduction (39). Intervention implications are therefore framed as hypotheses for longitudinal or experimental evaluation rather than prescriptions derived from the cross-sectional estimates.

### 4.7 Parental education and sex-specific effects

The inverse parental-education trend among girls (OR = 0.92) but not boys (*p* = 0.588) is exploratory. The score assumes equal spacing between education categories, and the analysis did not report a formal interaction test. The pattern may reflect differences in health literacy, home food environment, or parental monitoring, but these explanations were not directly measured and should be tested in future studies (40, 42).

### 4.8 Strengths and limitations

Strengths include the large school-based analytic sample, use of the Chinese WS/T 586–2018 BMI classification standard, detailed sex- and grade-stratified estimates, and bootstrap assessment of the fitted indirect component. The revision also reports the questionnaire’s actual item wording and coding, quantifies record retention by grade and region, and distinguishes the numerical software output from causal interpretation.

Several limitations are important. First, the cross-sectional design does not establish temporal ordering and is vulnerable to reverse causation. Second, device use, physical activity, sleep, and parental education were derived from study-specific self-report items without documented psychometric validation; the dataset did not identify who entered each item or whether younger children received assistance, device use did not distinguish academic from recreational purposes, and activity frequency did not measure guideline-level MVPA. Third, the height/weight measurement protocol could not be verified. Fourth, 46.9% of available records were excluded; the invitation denominator and true questionnaire response rate were unavailable, most excluded records lacked device-use data, and included and excluded records differed on available characteristics, so selection bias remains possible. Fifth, 73.3% of the analytic sample came from Daqing, and sampling probabilities were unavailable; the findings should not be generalized to all school-aged children in Heilongjiang Province. Sixth, the physical-activity associations were small and their clinical importance is uncertain. Seventh, the screen-use estimate varied with exposure specification, physical-activity adjustment, and clustered versus conventional uncertainty. Eighth, the ordinal activity, sleep, and education scores impose spacing assumptions. Ninth, diet, family lifestyle, geographic context, and other sedentary behaviors may confound the fitted pathways (43). Finally, the software labels ACME and ADE do not convert a cross-sectional decomposition into causal evidence.

## 5 Conclusion

Among the 14,095 participants in the original complete-case analysis, overweight/obesity prevalence was 38.2%. Male sex, younger grade level, a lower physical-activity frequency score, and a lower approximate sleep-duration score were associated with higher odds, although the activity association was modest. These findings describe the analyzed participating-school sample, which was concentrated in Daqing, and should not be interpreted as province-wide prevalence or effect estimates.

The unchanged exploratory decomposition estimated an indirect probability difference of 0.0011 and a descriptive indirect proportion of 24.35% through physical-activity frequency, while the direct probability difference was 0.0033. However, the added activity-adjusted continuous logistic model did not show a statistically significant independent association. The decomposition is therefore treated as an exploratory cross-sectional statistical pathway, not proof of mediation.

The categorical model, harmonized continuous models, and bootstrap decomposition did not give uniformly significant evidence for an independent device-use association. This model dependence is a central qualification. Longitudinal studies using validated or objective behavioral and anthropometric measures are needed to establish temporal ordering.

For practice, regular physical activity and balanced daily routines remain appropriate general health priorities. The current data do not justify claiming that screen-time reduction alone will cause a material reduction in obesity risk.

## Data Availability

The raw data supporting the conclusions of this article will be made available by the authors, without undue reservation.
