# Supplementary material for: Screen time, physical activity, and overweight/obesity among school-aged children and adolescents in Heilongjiang Province, China: a cross-sectional study
Source: Front Public Health. 2026 Oct 28;14:1959738. doi: 10.3389/fpubh.2026.1959738 (PMC13752090; doi:10.3389/fpubh.2026.1959738)
Supplement: Supplementary file 1 [file Supplementary_file_1.docx]

# Supplementary material

Supplementary Table S1. Questionnaire items and analytical coding retained in the original analysis

| Construct | Questionnaire content and respondent | Original analytical coding and limitation |
| --- | --- | --- |
| Daily electronic-device use | Student-facing section; average total daily electronic-device use | Four categories in submitted logistic models; continuous hours/day in added sensitivity models and decomposition |
| Physical-activity frequency | Student-facing section; average exercise frequency, including physical education classes | Approximate weekly-frequency score: 0, 0.5, 1, 3, 7 |
| Nighttime sleep duration | Student-facing section; five ordered duration categories | Approximate hour score used in the original analysis |
| Parental education | Student-facing family section; five education categories for each parent | Higher parental category, coded 1-5 as an ordinal trend |
| Height and weight | Questionnaire fields in cm and kg | BMI calculated; measurement protocol unavailable |
| Purpose/timing detail | Not collected by the electronic-device item | Academic/recreational and weekday/weekend use cannot be separated |
| Respondent and validation | Students were instructed to complete the survey; a later section was separately labelled for parents | Item-level respondent or assistance was not recorded; no reliability or validity assessment was documented for these study-specific items |

| Variable | Category/statistic | Excluded | Included | p-value |
| --- | --- | --- | --- | --- |
| Age (years) | Median [IQR] | 12.00 [10.00, 13.00] | 13.00 [12.00, 14.00] | <0.001 |
| Electronic-device use (hours/day) | Median [IQR] | 1.00 [0.50, 2.00] | 1.17 [0.50, 2.50] | <0.001 |
| Sex | Female | 6,259 (50.3%) | 7,148 (50.7%) | 0.516 |
|  | Male | 6,181 (49.7%) | 6,947 (49.3%) |  |
| Grade level | Senior high school | 1,842 (15.2%) | 3,574 (25.4%) | <0.001 |
|  | Junior high school | 4,067 (33.6%) | 7,198 (51.1%) |  |
|  | Upper primary school | 6,021 (49.7%) | 3,081 (21.9%) |  |
|  | Lower primary school | 183 (1.5%) | 242 (1.7%) |  |
| Region | Daqing | 7,827 (62.9%) | 10,332 (73.3%) | <0.001 |
|  | Yichun/Wangkui | 4,076 (32.8%) | 3,484 (24.7%) |  |
|  | Heihe | 537 (4.3%) | 279 (2.0%) |  |

Supplementary Table S2. Available-characteristic comparison of excluded and included records (included n = 14,095; excluded n = 12,440)

Percentages use available observations within each group. Differences indicate that complete-case selection should not be assumed to be completely random; the comparison does not eliminate residual selection bias.

Supplementary Table S3. Harmonised continuous electronic-device-use sensitivity models (n = 14,095)

| Continuous electronic-device-use model | OR per 1 hour/day (95% CI) | p-value |
| --- | --- | --- |
| Without physical-activity score; conventional SE | 1.019 (1.002-1.035) | 0.025 |
| With physical-activity score; conventional SE | 1.014 (0.998-1.031) | 0.085 |
| Without physical-activity score; school-clustered robust SE | 1.019 (0.999-1.039) | 0.069 |
| With physical-activity score; school-clustered robust SE | 1.014 (0.995-1.035) | 0.156 |

All models adjusted for sex, grade level, approximate sleep-duration score, highest parental-education ordinal score, and geographic region. Models labelled 'with physical-activity score' additionally adjusted for the original physical-activity frequency score. The three categorical screen-use terms in the submitted model were not jointly significant (conventional Wald p = 0.866; school-clustered Wald p = 0.851). SE, standard error; OR, odds ratio; CI, confidence interval.

Supplementary Table S4. Analytic-record retention within available grade and region categories

| Domain | Category | Included | Excluded | Available total | Retained (%) |
| --- | --- | --- | --- | --- | --- |
| Grade | Lower primary school | 242 | 183 | 425 | 56.9 |
| Grade | Upper primary school | 3,081 | 6,021 | 9,102 | 33.8 |
| Grade | Junior high school | 7,198 | 4,067 | 11,265 | 63.9 |
| Grade | Senior high school | 3,574 | 1,842 | 5,416 | 66.0 |
| Region | Daqing | 10,332 | 7,827 | 18,159 | 56.9 |
| Region | Yichun/Wangkui | 3,484 | 4,076 | 7,560 | 46.1 |
| Region | Heihe | 279 | 537 | 816 | 34.2 |

Retained (%) = included/(included + excluded) among records with the category available. Grade was unavailable for 327 excluded records. The table cannot estimate questionnaire response rates because the numbers initially invited and returning no questionnaire were not available.

Supplementary Figure S1. Prevalence of overweight/obesity stratified by sex and grade level among school-aged children and adolescents in the analytic sample (n = 14,095).

Supplementary Figure S2. Forest plot of sex-stratified multivariable logistic regression estimates. The original odds ratios and 95% confidence intervals are retained; variable labels reflect the questionnaire-derived scores and total daily electronic-device use.
